# Intestinal epithelial suppressor of cytokine signaling 3 enhances microbial-induced inflammatory tumor necrosis factor-α, contributing to epithelial barrier dysfunction

**DOI:** 10.1152/ajpgi.00214.2014

**Published:** 2014-11-06

**Authors:** Imtiyaz Thagia, Elisabeth J. Shaw, Emily Smith, Kathryn J. Else, Rachael J. Rigby

**Affiliations:** ^1^Division of Biomedical and Life Sciences, Faculty of Health and Medicine, Lancaster University, Lancaster, UK; and; ^2^Faculty of Life Sciences, Manchester University, Manchester, UK

**Keywords:** SOCS3, TNF-α, TNFR2, epithelium

## Abstract

A single layer of intestinal epithelial cells (IEC) lines the entire gastrointestinal tract and provides the first line of defense and barrier against an abundance of microbial stimuli. IEC homeostasis and repair are mediated through microbe-sensing Toll-like receptor (TLR)-induced inflammatory pathways. Increasing evidence supports a role of suppressor of cytokine signaling 3 (SOCS3) as a modulator of IEC turnover, balancing controlled repair and replenishment with excessive IEC proliferation predisposing to dysplasia and cancer. Our data indicate that SOCS3 can limit microbial-induced IEC repair, potentially through promoting tumor necrosis factor-α (TNF-α) and limiting TNFR2 expression. Activation of TLR5 signaling pathways, compared with other TLR, increases TNF-α mRNA in a dose-dependent manner and SOCS3 enhances TLR5-induced TNF-α. We also show that flagellin promotes transcription of TNFR2 and that SOCS3 limits this expression, presenting a mechanism of SOCS3 action. Our data also support the role of microbial ligands in epithelial wound healing and suggest that a functional consequence of increased TNF-α is reduced wound healing. These results provide further evidence to support the regulatory role of epithelial SOCS3 in intestinal health and suggest that the increased expression of SOCS3 observed in IBD may serve to perpetuate “inflammation” by promoting TNF-α production and limiting epithelial repair in response to commensal microflora.

the homeostatic influence of microbiota on intestinal epithelial cell (IEC) turnover occurs in an actively regulated environment, dictated by signals between the microbiota, IEC, and IEC-conditioned components of the immune system (2, 23). IEC are able to converse with microbes in the colon via a cohort of pattern recognition receptors including Toll-like receptors (TLR) (1). TLR signaling is paramount in driving intestinal tissue repair and regeneration (19), as evidenced by germ-free mice and mice deficient in MyD88 having an impaired ability to repair intestinal injury (20). TLR-2, TLR-4, and TLR-5 knockout mice, though not to the same extent as MyD88^−/−^ mice, have a reduced capacity to repair colonic mucosa and reduced barrier function, with TLR5 knockout mice developing spontaneous colitis (20, 27). TLR signaling pathways are important activators of NF-κB, driving the expression of a number of “proinflammatory” genes such as TNF-α, IL-6, and IL-8 essential for tissue repair and maintenance of barrier function (26). However, persistent activation of such pathways can lead to certain pathologies, including chronic inflammation characteristic of inflammatory bowel disease (IBD) and some cancers. Failure to regulate microbial interactions is implicated in the onset of IBD, where damage to the intestinal barrier is characteristic of chronic relapse and remittance of inflammation.

Suppressor of cytokine signaling-3 (SOCS3), an endogenous modulator of IEC turnover, is upregulated in IBD (15, 24). SOCS3 is a tumor suppressor in the intestine (21), as IEC-targeted deletion of SOCS3 promotes tumor burden in the colon, with methylated silencing of SOCS3 shared in multiple tumor types (12, 17, 21). SOCS3 is also a potent suppressor of proliferation in both transformed and nontransformed IEC lines, supporting a role in mediating IEC turnover, ostensibly due to regulation of “inflammatory” signaling, for example by limiting TNF-α-induced NF-κB translocation and IL-6-induced STAT3 phosphorylation (9, 12, 17, 21).

It has been well documented that levels of TNF-α are elevated in serum and intestinal mucosa of patients with IBD with neutralization of TNF-α, the basis of anti-TNF therapy, being associated with improved health, particularly in patients with Crohn's disease (25). TNF-α signals through a family of receptors, including two transmembrane receptors, TNFR1 and TNFR2. TNF-α signaling through TNFR1 and TNFR2 induces the activation of the transcription factors AP-1 and NF-κB, linked to cell proliferation, survival, and/or apoptosis (3). TNFR2 is upregulated in IBD and in models of inflammation-associated cancer (6, 16, 18), regarded to be in response to increased inflammatory cytokines in the mucosa, as TNF-α and IL-6 induce TNFR2 in colon cancer cells (9, 16). TNF-α is shown to be a major mediator of epithelial barrier dysfunction (7, 28, 29), and TNF-α-induced loss of intestinal epithelial barrier function requires both TNFR1 and TNFR2 signaling (8).

We propose that SOCS3, ostensibly an endogenous inhibitor of inflammatory signaling and proliferation, paradoxically promotes intestinal inflammation, possibly through limiting microbial-induced TNFR2 expression, enhancing TNF-α production and thus limiting epithelial barrier repair.

## MATERIALS AND METHODS

### 

#### Cell lines and culture.

SW480 (ECACC), human colorectal cancer IEC, were maintained in Leibovitz growth medium (GIBCO) supplemented with 10% fetal bovine serum (FBS), 50 units/ml of penicillin and streptomycin (Sigma), and 0.1 mg/ml sodium pyruvate. Caco-2 epithelial cell line (ECACC), was maintained in MEM (GIBCO) supplemented with 10% FBS, 50 units/ml of penicillin and streptomycin, and 1% MEM Non-Essential Amino Acids (GIBCO). Cells were allowed to differentiate under an atmosphere of 95% (vol/vol) air-5% CO_2_ at 37°C in a humidified incubator.

#### Generation of SOCS3-overexpressing cells.

The mammalian expression vector pIRESneo, with and without the full-length human SOCS3 cDNA and containing the Gentacin antibiotic resistance cassette, was purchased from Epoch Life Science. Plasmids were linearized with Ahd1 (NEB no. R0584S) prior to ethanol precipitation. For transfection, cells were seeded at a concentration of 1 × 10^6^/ml and incubated with Lipofectamine 2000 (Invitrogen) and plasmid DNA, with and without the full-length human coding SOCS3 cDNA, referred to as SOCS3^hi^ and SOCS3^norm^, respectively, according to manufacturer's instructions. G418 antibiotic, at varying concentrations, was used to select transfected IEC. Medium containing 0.4 mg/ml G418 antibiotic was used to maintain transfected IEC during culture. To validate overexpression of SOCS3, protein and mRNA levels were measured in SOCS3^norm^ and SOCS3^hi^ IEC. SOCS3^hi^ depicted a 12 ± 2-fold increase in SOCS3 mRNA compared with SOCS3^norm^ IEC. Immunoblotting showed a 1.3 ± 0.1-fold increase in SOCS3 protein expression compared with SOCS3^norm^ IEC (data not shown).

#### Stimulation with TLR ligands.

SOCS3^norm^ and SOCS3^hi^ IEC at a concentration of 2 × 10^5^/ml were allowed to adhere for 3 days, with serum deprivation overnight before treatment. IEC were treated with polyinosinic:polycytidylic acid [Poly(I:C)] (InvivoGen), LPS *Escherichia coli* (Sigma), flagellin *Salmonella typhimurium* (InvivoGen) or *Trichuris muris* excretory/secretory protein (ES, gift from K. Else) at varying concentrations and times. Supernatant was collected and stored at −80°C for ELISA assays and IEC lysed with either TRI Reagent (for RNA, Sigma) or lysis buffer (Western blot) or fixed for immunohistochemistry.

#### qRT-PCR.

Total RNA from each sample was extracted by using TRI Reagent according to the manufacturer's instructions. The concentration and purity of RNA was determined by use of the NanoDrop 2000c (Thermo Scientific). Reverse transcriptase (RT) was performed by using the Enhanced Avian RT First Strand Synthesis Kit (Sigma) with Anchored oligo(dT) (Thermo Scientific) and PCR nucleotide mix (Fisher) to generate cDNA by using 2 μg of RNA. TNF-α validated primers were purchased from Primerdesign, reconstituted, and used as specified in the supplemental data sheet. The TNFR2, SOCS3, and housekeeping gene RPLPO primers were designed and checked for specificity by use of BLAST search from the National Center for Biotechnology Information (NCBI). Forward and reverse primers (Sigma) were first reconstituted in autoclaved Milli-Q water to 100 μM according to the supplied data sheet before being used to amplify TNFR2, SOCS3, and RPLPO cDNA. The primer sequences used to amplify TNFR2 (148 bp) were Forward: GAGTGGTGAACTGTGTCATGA and Reverse: GAGCTCGGCGCTGTGATC; SOCS3 (119 bp) Forward: TCGATTCGGGACCAGCCCCC and Reverse: TGCTGTGGGTGACCATGGCG; and RPLPO (142 bp) Forward: GCAATGTTGCCAGTGTCTG and Reverse: GCCTTGACCTTTTCAGCAA.

#### qRT-PCR analysis.

Real-time PCR was performed by using the SYBR Green JumpStart Taq ReadyMix (Sigma) and the C1000 Real-Time Thermocycler (Bio-Rad). Cycling conditions were 94°C for 2 min, 40 × 94°C for 15 s, and 60°C for 1 min. Gene expression was calculated using the *R* = 2^(−ΔΔCt)^ method, where changes in C_t_ values for the gene of interest were normalized relative to the “housekeeping gene” RPLPO. In all experiments, gene expression was expressed as fold change relative to no-treatment control. Real-time PCR reactions were carried out in duplicate and replicated in a minimum of three independent experiments.

#### Western blot.

Cells were lysed in 100 μl of lysis buffer (150 nM NaCl, 50 mM Tris·HCl, 2 mM EDTA, 1 mM NaVO_4_, 1 mM NaF, and 1% Nonidet P-40) supplemented with protease and phosphatase inhibitor cocktails. Samples were spun at 4°C at 12,000 rpm for 10 min, and Bradford reagent (Sigma) was used to determine protein concentration. We boiled 25 μg of protein with 4× sample buffer (Invitrogen) and 2-mercaptoethanol (Sigma) at 95°C for 10 min and performed SDS-PAGE. Proteins were transferred onto nitrocellulose membrane (Macherey-Nagel) by using the Trans-Blot Turbo Transfer system (Bio-Rad). Membranes were blocked with 3% BSA in TBS for 1 h. Primary antibodies pSTAT3 (Santa Cruz no. sc-8001-R), total STAT3 (Santa Cruz no. sc-8019), p65 (Cell Signaling no. 3037S), β-actin (Cell Signaling no. 4967), and horseradish peroxidase-conjugated secondary antibody (Santa Cruz nos. sc-2030 and sc-2031) were used and their presence was quantified by use of the ChemiDoc XRS imaging system (Bio-Rad). The densitometry signal from loading control β-actin was used to normalize signal from p65 and pSTAT3 bands.

#### Immunocytochemistry.

IEC were seeded onto coverslips at 1 × 10^5^ IEC/well and allowed to adhere overnight. Following flagellin treatment, medium was removed and coverslips were washed three times in PBS. IEC were fixed in ethanol:methanol for 10 min at −20°C, blocked in 2% BSA for 30 min at room temperature, and incubated in 0.5 μg/ml of Anti-Human sTNF Receptor Type II (Peprotech 500-P168) at 4°C overnight. Secondary antibody Alexa Fluor 488 donkey anti-rabbit IgG (Life Technologies, 1:200) was used and cells were counterstained with propidium iodide (Life Technologies, P3566) 1:3,000 in PBS for 1 min, coverslipped, and mounted onto slides to be viewed via the Zeiss LSM 510 Meta Confocal Microscope.

#### Wound healing assay.

Wound healing assay was performed by a modified method as described by Han and colleagues (11). Caco-2 cells (2 × 10^5^ cells/well) were seeded at 80–90% confluence in a 24-well plate and allowed to form monolayers for 7 days at 37°C. After 7 days, linear wounds were made with a sterile 10-μl plastic pipette tip. Wells were washed three times in PBS to remove displaced cells and treated as required. Images of wounds were obtained using the confocal microscope (Leica DMIRE2 inverted microscope) at 10 × magnification by using standard protocols at the predetermined location at 0 h and 48 h after wounding. The total area of the wound was measured with ImageJ and data were calculated as % wound healed vs. 0 h. Medium and respective treatments were replaced after 24 h to remove dislodged cells, replenish nutrients, and restore treatment levels.

#### Statistical analysis.

Experiments were repeated multiple times and data were pooled together. To determine statistical significance, ANOVA was performed with two-sided Dunnett's or Tukey's post hoc tests where appropriate.

## RESULTS

### 

#### TLR5 ligation promotes TNF-α in a dose-dependent manner.

**Fig. 1.**
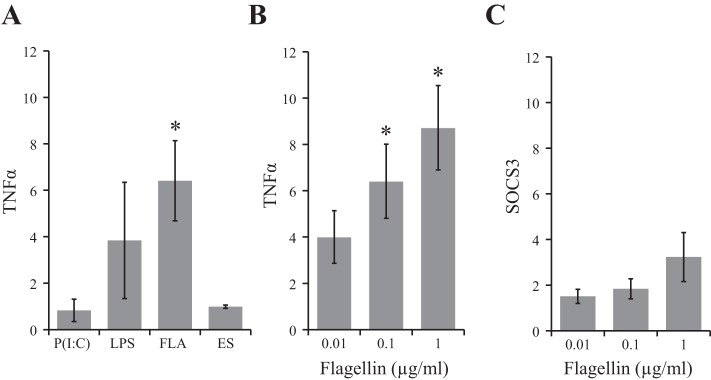
Fold increase in tumor necrosis factor-α (TNF-α; *A* and *B*) and suppressor of cytokine signaling 3 (SOCS3; *C*) mRNA following 2 h stimulation with microbial products relative to no treatment. *A*: polyinosinic:polycytidylic acid [Poly(I:C); P(I:C); 0.1 μg/ml], LPS (0.1 μg/ml), flagellin (FLA; 0.1 μg/ml), and *Trichuris muris* excretory/secretory protein (ES; 0.1 mg/ml) were added to the culture medium. Flagellin was the only Toll-like receptor (TLR) ligand shown to significantly promote TNF-α mRNA (*n* = 5). *B*: flagellin promoted TNF-α expression in a dose-dependent manner (*n* = 5). *C*: flagellin did not induce SOCS3 mRNA relative to no-treatment controls (No Tx) (*n* = 6). *P ≤ 0.05 vs. No Tx.

To determine the effect of TLR ligation on IEC TNF-α mRNA, SW480 IEC were stimulated with TLR agonists: Poly(I:C) (TLR3), flagellin (TLR5), LPS (TLR4), or *T. muris* ES for 2 h. TNF-α mRNA was normalized to RPLP0 mRNA, and results were displayed as fold change relative to no treatment. Data illustrate that flagellin was the only TLR ligand shown to significantly promote TNF-α mRNA expression (6.41 ± 1.7, *P* ≤ 0.05) (Fig. 1*A*). When IEC were treated with varying concentrations of flagellin for 2 h, a dose-dependent increase in TNF-α mRNA was observed; 0.1 μg/ml and 1 μg/ml of flagellin significantly promoted TNF-α transcription (6.4 ± 1.6, *P* ≤ 0.03; 8.7 ± 1.8, *P* ≤ 0.01, respectively) compared with no-treatment control (Fig. 1*B*). Treatment with the lowest concentration of flagellin (0.01 μg/ml) showed a nonsignificant increase in TNF-α mRNA expression in IEC (4.0 ± 1.1, *P* = 0.06), providing further support that the response is dose dependent. SOCS3 mRNA was not significantly changed following flagellin treatment compared with no-treatment control (Fig. 1*C*).

#### SOCS3 enhances TLR5-induced TNF-α mRNA.

**Fig. 2.**
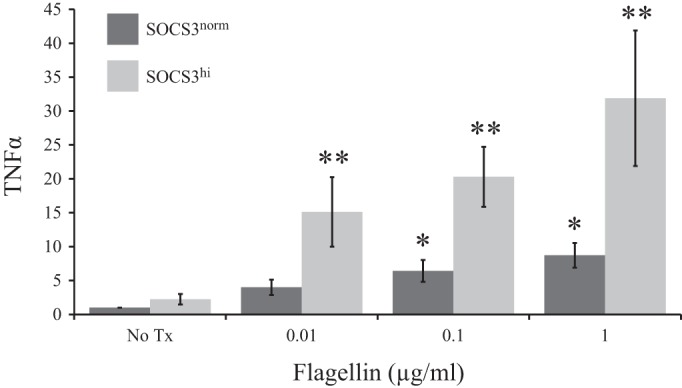
SOCS3 overexpression enhanced flagellin-induced TNF-α mRNA in a dose-dependent manner. Bar chart depicts fold increase in TNF-α mRNA, in Control (SOCS3^norm^) and SOCS3 overexpressing (SOCS3^hi^) intestinal epithelial cells (IEC) relative to no-treatment (No Tx) SOCS3^norm^, following 2-h flagellin treatment. *P ≤ 0.05 vs. No Tx; **P ≤ 0.05 SOCS3^norm^ vs. SOCS3^hi^.

Flagellin-induced TNF-α mRNA was measured in SOCS3^hi^ and compared with control SOCS3^norm^ IEC. As previously observed, TNF-α was enhanced in a dose-dependent manner in SOCS3^norm^ cells (0.01 μg/ml: 15.1 ± 5.1, *P* ≤ 0.04; 0.1 μg/ml: 21.3 ± 4.3, *P* ≤ 0.01 and 1 μg/ml: 31.9 ± 9.9, *P* ≤ 0.03) compared with no-treatment control (Fig. 2). Surprisingly, flagellin-induced TNF-α was enhanced in IEC overexpressing SOCS3. Treatment of SOCS3^hi^ with the lowest concentration of flagellin (0.01 μg/ml) resulted in a 3.8-fold increase in TNF-α mRNA compared with SOCS3^norm^. Fold increases of 3.2 and 3.7 were observed in SOCS3^hi^ vs. SOCS3^norm^ after treatment with 0.1 and 1 μg/ml of flagellin, respectively (*P* ≤ 0.05). However, at 2 h SOCS-mediated increases in TNF were not detectable in the supernatant (ELISA data, not shown).

#### SOCS3 overexpression does not limit flagellin-induced pSTAT3 or NF-κB.

**Fig. 3.**
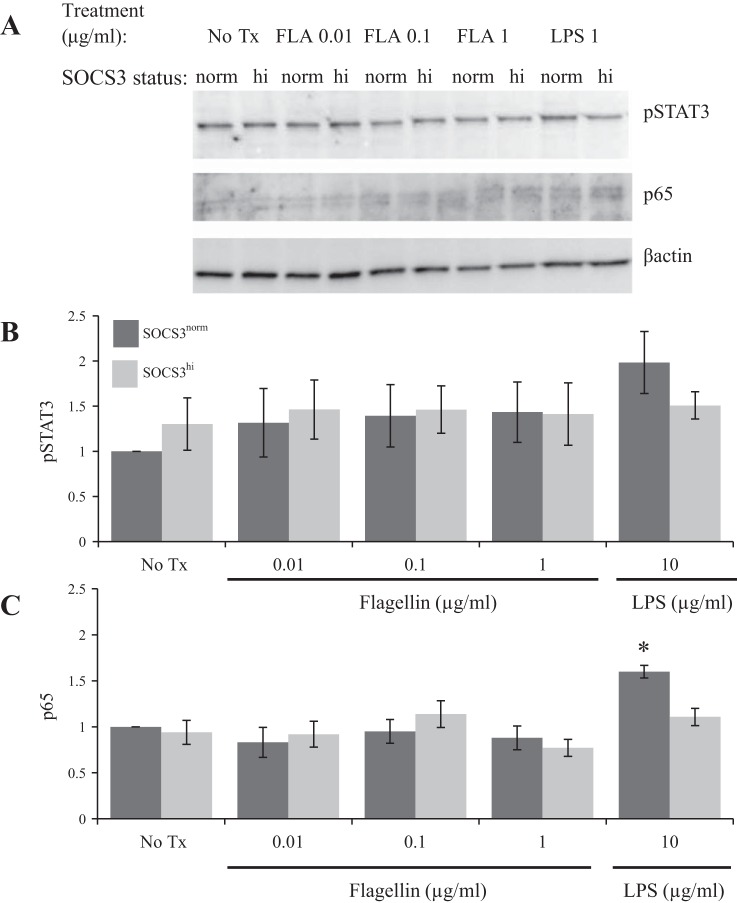
Flagellin treatment did not promote STAT3 phosphorylation or NF-κB p65 activation relative to No Tx. *A*: representative Western blot of pSTAT3 and p65 following 2-h flagellin or LPS stimulation at varying concentrations. *B*: average relative pSTAT3 and p65 (*n* = 3) expression following 2-h stimulation. SOCS3 limited LPS (10 mg/ml)-induced p65 *P ≤ 0.05 vs. No Tx. norm, Normal; hi, high.

To assess whether the impact of SOCS3 on TNF-α was due to differential phosphorylation of STAT3 and/or dissociation of NF-κB p65, these transcription factors were assessed by Western blot (Fig. 3*A*) in SOCS3^norm^ and SOCS3^hi^ treated with varying concentrations of LPS and flagellin for 2 h. As expected, LPS (10 μg/ml) induced an increase in NF-κB p65 (1.6 ± 0.07, *P* ≤ 0.04) in SOCS3^norm^ vs. no-treatment control and SOCS3 overexpression inhibited LPS-induced p65 (Fig. 3*B*). Flagellin treatment had no significant impact on either pSTAT3 or p65 vs. no treatment. To conclude, SOCS3 did not appear to mediate its effects on flagellin-induced TNF-α through differential activation of pSTAT3 or NF-κB.

#### TLR5 ligation induces transient downregulation of TNFR2.

**Fig. 4.**
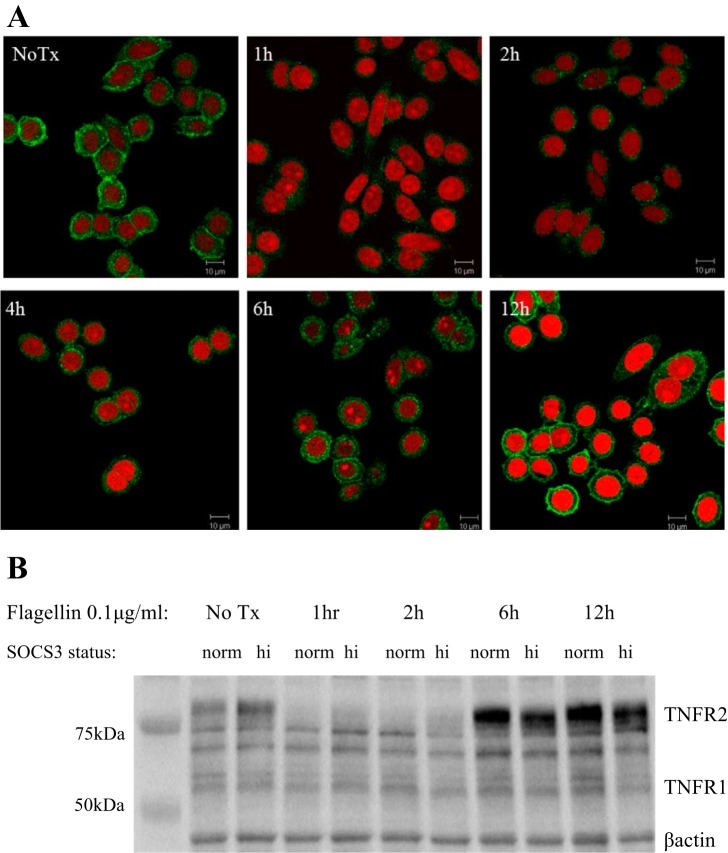
TNFR2 was rapidly downregulated following flagellin treatment. Protein expression levels returned to baseline by 12 h posttreatment. *A*: representative photomicrographs of TNFR2 following flagellin treatment. Immunocytochemistry of IEC, untreated and following 1, 2, 4, 6, or 12 h flagellin treatment (0.1 μg/ml). Green, TNFR2; red, propidium iodide. *B*: Western blot, confirming TNFR2 immunochemistry staining and depicting TNFR1 expression following varying time points of flagellin treatment.

We performed immunocytochemistry and Western blots to assess how TLR5 stimulation impacted on IEC TNFR2 protein expression. TNFR2 expression appeared reduced within 1 h at the cell surface receptor level and was regained to baseline by 6 h after flagellin treatment (Fig. 4*A*). SOCS3 overexpression may delay TNFR2 return to baseline levels, but expression appeared equivalent to that of control cells 12 h following treatment. Flagellin did not appear to impact of TNFR1 expression (Fig. 4*B*).

#### SOCS3 limits TLR5- and TLR3-induced increases in TNFR2 transcription.

**Fig. 5.**
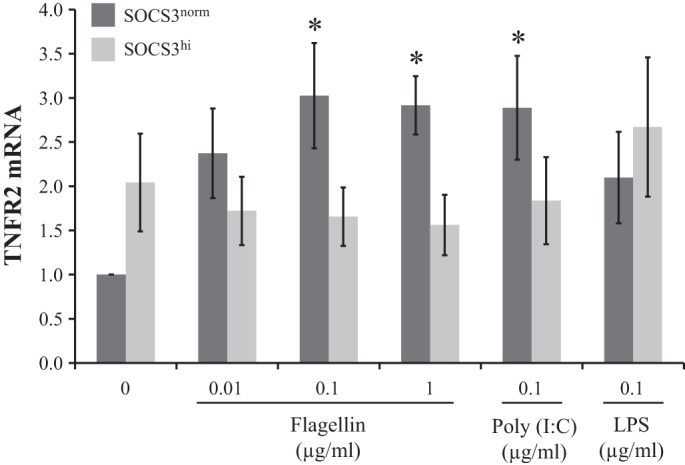
SOCS3 limits flagellin (TLR5)- and Poly(I:C) (TLR3)-induced TNFR2 expression. Bar chart indicates relative TNFR2 mRNA following 2-h TLR ligand treatment of SOCS3^norm^ and SOCS3^hi^ (*n* = 4) relative to No Tx. *P ≤ 0.05 vs. No Tx.

To establish whether SOCS3 inhibits microbial-induced as well as cytokine-induced TNFR2, mRNA levels were assessed in response to various TLR ligands; SOCS3 overexpressing IEC (SOCS3^hi^) and control (SOCS3^norm^) IEC were treated with flagellin, Poly(I:C), or LPS for 2 h (Fig. 5). Most concentrations of flagellin (0.1 and 1 μg/ml) and Poly(I:C) (0.1 μg/ml) were shown to significantly enhance TNFR2 mRNA vs. no-treatment control, by 3.0 ± 0.6, 2.9 ± 0.3, and 2.9 ± 0.6-fold, respectively (Fig. 5), presumably accounting for the replenishment of protein expression observed in Fig. 4. However, upregulation of TNFR2 mRNA was not observed in SOCS3^hi^ IEC in response to either TLR5 or TLR3 ligation, indicating that SOCS3 inhibits microbial-induced TNFR2 at the transcription level.

#### SOCS3 limits microbial-induced wound healing.

**Fig. 6.**
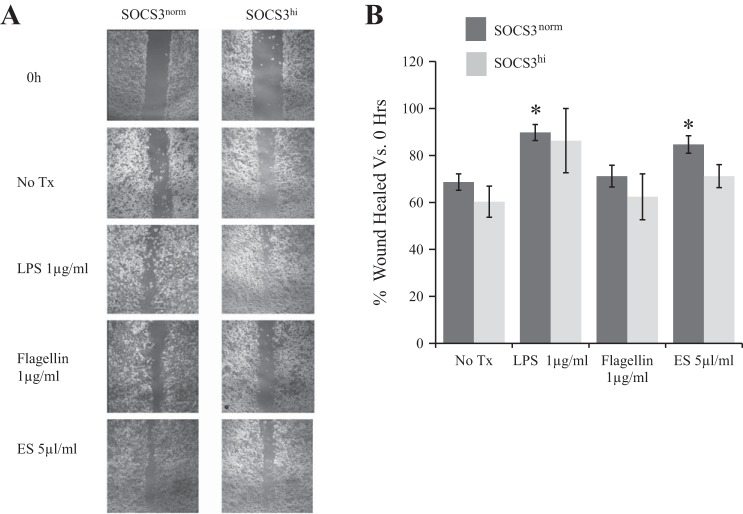
*A*: Representative photomicrographs of TLR-induced epithelial wound repair in SOCS3^norm^ and SOCS3^hi^ IEC. Wounds measured at 48 h following injury. *B*: flagellin does not promote epithelial wound repair compared with other microbial products (*n* ≥ 3). Bar chart indicates percentage wound healed at 48 h vs. 0 h. *P ≤ 0.05 vs. No Tx.

A Caco-2 model of epithelial wound repair was used to determine how microbial products impact on would healing. LPS and ES treatment promoted wound healing above that of no-treatment controls, 21 ± 3.4 and 16 ± 3.7%, respectively (Fig. 6), whereas flagellin did not promote wound healing (71 vs. 69% in no-treatment control). SOCS3 overexpression inhibited the wound healing effect of both LPS and ES.

## DISCUSSION

We have shown that SOCS3 promotes TLR5-induced increases in TNF-α, an important pathological cytokine in IBD. TNF-α disrupts intestinal epithelial barrier function (29) and emerging evidence suggests that flagellin is a major driver of TNF-α in pathological inflammation (5). Furthermore, SOCS3 limits microbial-induced transcription of TNFR2, providing further mechanistic support for its role in regulating homeostasis as well as a tumor suppressor role in the intestine (21). It is well established that microbial products drive epithelial repair (20), and accumulating evidence suggest that dysregulated signaling, linked to increased TNF-α (29), may impair epithelial replenishment and repair. Indeed, the observation that flagellin does not promote wound healing, unlike alternative microbial products tested, may be a functional consequence of increased TLR5-driven TNF-α, with increased SOCS3, observed in chronic intestinal inflammation (15, 24), exacerbating pathological TNF-α secretion. Mechanistically, the limiting effect of SOCS3 on TNFR2 expression could account for the observed TLR5-induced increases in TNF-α. TNF-α regulates the expression of its TNFR2 receptor (4, 13), presumably to limit excessive signaling, but because SOCS3 limits TNFR2 upregulation in response to flagellin, delaying the negative feedback loop, IEC may transcribe more TNF-α to compensate, resulting in the chronic inflammation characteristic of IBD. It remains to be determined whether SOCS3 can drive the increases in TNF-α independent of downregulating TNFR2, or indeed whether increased TNF-α transcription is maintained, but it is likely that these two mechanisms are interrelated. Alternatively, SOCS3 may promote posttranscriptional degradation of TNF-α, through either proteasome (10) or autophagy (14) mechanisms, and the subsequent cellular response is to drive more TNF-α transcription to compensate for the lack of autocrine feedback.

Our findings are in accordance with studies demonstrating that SOCS3 limits inflammatory cytokine-induced TNFR2 in colonic IEC (9). The human TNFR2 promoter contains 2 consensus STAT binding sites (22), which may account for the ability of SOCS3 to limit TNFR2 transcription. However, the impact of flagellin on TNFR2 is unlikely to be due to inflammatory cytokines in our system, since at 2 h posttreatment the increased TNF-α has little time to impact in an autocrine manner and our lack of detectable TNF-α in the supernatant supports this assumption. Therefore we initially hypothesized that SOCS3 is likely to directly impact on flagellin-induced transcription factor binding; however, SOCS3 does not appear to be limiting NF-κB or STAT3 activation in response to TLR5 ligation. In our system flagellin treatment did not result in increased p65, but SOCS3 did limit p65 following LPS treatment. Flagellin itself does not significantly change SOCS3 mRNA expression, at least at the 2-h time point studied, supporting the finding that pSTAT3 is not involved in TLR5 signaling of IEC. It remains to be determined whether SOCS3 mediates its impact on TNF-α transcription through other transcription factors. Our results also illustrate the varied complexity of microbial signaling at the IEC interface. For example, SOCS3 does appear to limit LPS-induced p65 (Fig. 3*B*) and flagellin does not appear to impact on TNFR1 expression (Fig. 4*B*).

## GRANTS

This work was funded by an MRC New Investigator Research Grant (G1100211).

## DISCLOSURES

No conflicts of interest, financial or otherwise, are declared by the author(s).

## AUTHOR CONTRIBUTIONS

I.T., E.J.S., and E.S. performed experiments; I.T. and R.J.R. analyzed data; I.T. and R.J.R. interpreted results of experiments; I.T. prepared figures; I.T. drafted manuscript; E.J.S., E.S., and R.J.R. approved final version of manuscript; K.J.E. and R.J.R. edited and revised manuscript; R.J.R. conception and design of research.
